# Relationship between Mast Cells and the Colitis with Relapse Induced by Trinitrobenzesulphonic Acid in Wistar Rats

**DOI:** 10.1155/2009/432493

**Published:** 2009-05-11

**Authors:** Ana Carolina Luchini, Déborah Mara Costa de Oliveira, Cláudia Helena Pellizzon, Luiz Claudio Di Stasi, José Carlos Gomes

**Affiliations:** ^1^Department of Pharmacology, Institute of Biosciences, São Paulo State University (UNESP), 18618-000 Botucatu, SP, Brazil; ^2^Department of Morphology, Institute of Biosciences, São Paulo State University (UNESP), 18618-000 Botucatu, SP, Brazil

## Abstract

The present study aimed to clarify the role of mast cells in colitis with relapse induced in Wistar rats by trinitrobenzenosulphonic acid. Colitis induction increased the histamine concentration in the colon, which peaked on day 26. The number of mast cells, probably immature, was ten times higher on day 8. Different from animals infected with intestinal parasites, after colitis remission, mast cells do not migrate to the spleen, showing that mast cell proliferation presents different characteristics depending on the inflammation stimuli. Treatment with sulfasalazine, doxantrazole, quercetin, or nedocromil did not increase the histamine concentration or the mast cell number in the colon on day 26, thereby showing absence of degranulation of these cells. In conclusion, although mast cell proliferation is associated with colitis, these cells and their mediators appear to play no clear role in the colitis with relapses.

## 1. Introduction

During mast cell degranulation, a variety of mediators are released including histamine, prostaglandin D2, leukotriene C4, platelet activating factor, heparin, and neutral proteases. These cells may be involved in the etiology of inflammatory diseases through their activation and degranulation [1], including inflammatory bowel disease (IBD) and its main clinical manifestations such as ulcerative colitis (UC) [2] and Crohn's disease (CD) [3]. IBD is characterized by the development of chronic intestinal inflammation with alternating periods of remission and active inflammatory process. The etiology of IBD remains unknown, although it is believed to involve several interactions with immune, environmental, and genetic factors [4]. Histamine the main mast cell mediator—known to increase vascular permeability, leukocyte infiltration, and smooth muscle contraction—has been suggested as participating in intestinal inflammation [5]. In fact, rectal biopsies from inflamed areas in IBD release histamine spontaneously [6]. 

The mast cell participation in the intestinal mucosa of patients with either ulcerative colitis or Crohn's disease is controversial; reports have shown mast cell numbers being increased [7], decreased [8], or stable between active IBD and control biopsies [9]. These discrepancies may arise from differences in measurement techniques (tissue fixation, staining, cell counting) or due to the course of the inflammatory process (active disease, remission, drug treatment) [10]. Indeed, evaluation of mast cell function in vivo is very complex because mast cells may release several mediators endowed with opposite biological effects [11] and also participate in fibrosis and the wound healing process [11–13]. All these findings have led to the hypothesis that mast cells are not only proinflammatory effector cells but also a regulatory component of the immune system with an active involvement in tissue repair [12–14].

In comparison with clinical research, experimental assays using different animal species may provide stable disease models with less variation and, given an adequate number of animals in the sample, appropriate statistical data analysis. Since animal studies simulate the clinical symptoms and/or pathogenesis, the data obtained from these studies are useful for evaluation of disease etiology. Studies of mast-cell-deficient Ws/Ws rats have shown that mast cells are not essential to the development of TNBS-induced colitis. However, these prior studies used rats with TNBS-induced colitis without the relapse, a common clinical manifestation in the human IBD, and performed evaluations only on days 7 and 14 after colitis induction [15].

In this way, the present study aimed to determine if mast cells and their mediators played any role in colitis with relapse induced by TNBS in Wistar rats. Also, we verified whether, during colitis remission, these mast cells tend to migrate to the spleen, similarly to what occurs in animals infected with bowel parasites [16, 17]. Moreover, we employed inhibitors of mast cell secretion (nedocromil [18], quercetin [19], and doxatranzole [20]) to verify whether the colon mast cells are activated in ulcerative colitis with relapse.

## 2. Materials and Methods

### 2.1. Animals

Specific pathogen-free (SPF) male Wistar rats (180–200 g) obtained from CEMIB-UNICAMP (Campinas, SP, Brazil) were housed in markrolon cages (5 rats per cage) and maintained in air-conditioned animal quarters with 12 hours light-dark cycle. Animals had free access to water and food. The experimental protocol used was approved by the Ethics Committee for Animal Experimentation of the Institute of Biosciences, UNESP, Botucatu, SP, Brazil.

### 2.2. Drugs and Solutions

Doxatranzole (Merck) was dissolved in 5% dimethyl sulfoxide (DMSO). Nedocromil (Sigma) and quercetin (Sigma) were dissolved in distilled water and sulfasalazine (Sigma) in 1% methylcelulose. The concentrations of DMSO and methylcellulose used do not interfere with spontaneous histamine release.

### 2.3. Induction of Ulcerative Colitis with Relapse

Colitis was induced by the method originally described by Morris et al. [21]. Animals fasted overnight and were anaesthetized with halothane. Under anesthesia, they received 10 mg of TNBS dissolved in 0.25 mL 50% ethanol (v/v) by means of a teflon cannula inserted 8 cm through the anus. Rats from noncolitic group received 0.25 mL of phosphate buffered saline. After 14 days, the animals received a second dose of 10 mg of TNBS in an attempt to mimic the relapses common in human IBD. After TNBS administration, rats were kept in a head-down position until they recovered from anesthesia, and then returned to their cage. Animal body weights and occurrence of diarrhoea (as detected by perianal fur soiling) for each group were recorded daily.

### 2.4. Experimental Design

Two experimental designs were adopted in the present study. In the first one, we assessed the peak histamine concentration, the mast cell number, the macroscopic features of colonic lesions, and the possible mast cell migration to the spleen. Based on these preliminary data, the second experiment was designed to evaluate the possible involvement of mast cells and histamine in the TNBS-induced colitis relapse. In the second experiment, the animals were treated with 25 mg/Kg/day of sulfasalazine (p.o.), 100 mg/Kg/day of nedocromil (i.p.), 5 mg/Kg/day of doxantrazole (i.p.), or 10 mg/Kg/day of quercetin (i.p.) for, 12 days after relapse induction. The treatments started 2 hours before the relapse induction and extended to the 26th day after the initial colitis induction, which corresponds to the peak histamine concentration. The parameters evaluated were the total tissue histamine concentration, the mast cell number, and the macroscopic features of colonic damage in colitis-relapsed animals.

### 2.5. Macroscopic Analysis

Every two days two group of animals (TNBS and saline group) were euthanized by an overdose of halothane, the colonic segments were obtained after laparotomy, and the eventual occurrence of adhesions between the colon and adjacent organs was noted. They were placed on an ice-cold plate, cleaned of fat and mesentery, blotted on filter paper, weighed, and their lengths measured under a constant load (2 g). The colon was longitudinally opened and scored for macroscopically visible damage on a 0–10 scale (Table 1) by two observers unaware of the treatment, according to the criteria described by Bell et al. [22].

### 2.6. Tissue Histamine Concentration

The total histamine amount in the colon was determined for each animal from all the experimental groups. Tissue samples (approximately 30–100 mg) to be used for evaluation of tissue histamine concentration were weighed, the boiled in 3 mL of 0.9% saline for 10 minutes. The supernatants were trasferred to clean tubes and stored at –20°C until analysis. The samples of the colon were analyzed, after the removal of proteins by perchloric acid (2%) by the fluorometric method of Shore et al. [23] using a modular automatic continuous flow system [24], with the omission of the extraction steps.

### 2.7. Microscopic Analysis

In the colitis relapse protocol, representative whole gut specimens were taken from a region of the inflamed colon corresponding to the adjacent segment to assess the gross macroscopic damage and were fixed in Alfac solution (alcohol 68%, acetic acid 5%, and formaldehyde 10%) for 24 hours. Cross-sections were selected and embedded in paraffin. Equivalent colonic segments were also obtained from the saline group. Sections of 10 *μ*m were obtained from different levels and stained with 0.5% toluidine blue (pH 0.5) [25]. The number of mucosal mast cells in 10 randomly selected high-power fields (×100) was counted, and the number per 10 fields was calculated. The samples were analyzed by light microscopy.

### 2.8. Statistical Analysis

All results are expressed as mean ± S.E.M. Differences were tested using one way analysis of variance (ANOVA) and post hoc least significance tests or by Student's *t*-test for unpaired samples. Nonparametric data (score) are expressed as median and interquartile range (*Q*_1_–*Q*_3_) and were analyzed with the Mann-Whitney U test or Kruskal-Wallis test. Differences between proportions were analyzed by the Fisher's exact test. Statistical significance was set at *P* < .05.

## 3. Results

**Figure 1:**
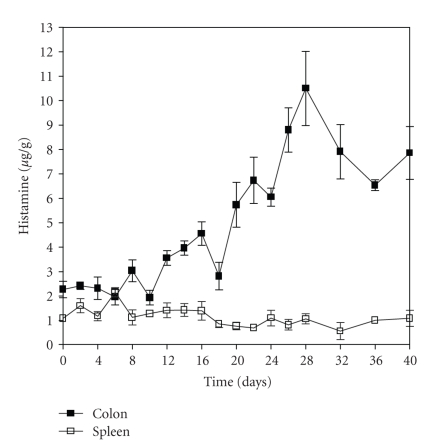
Histamine concentration in colon and spleen of Wistar rats with colitis induced by TNBS. The results are expressed as mean ± SEM (*n* = 6).

The histamine content profile in the colon of TNBS-treated Wistar rats did not change until the 10th day, when it started increasing, peaking around the 26th day, and remaining stable at least until day 40. The treatment did not change the histamine content (from days 0 to 40) in the spleen (Figure 1). 

**Figure 2:**
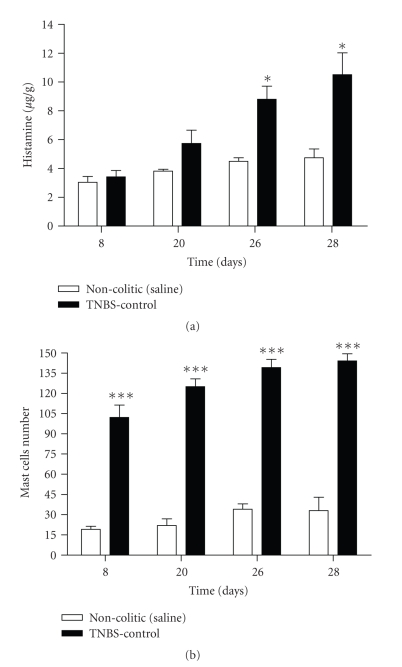
Histamine concentration and mast cells number in colon of Wistar rats with colitis induced by TNBS. The results are expressed as mean ± SEM (*n* = 5–7). *P < .05; ***P < .001 (by Student's *t*-test).

Although there was a significant increase (about 10 fold) in the mast cell number on days 8, 20, 26, and 28, the increase in the histamine content after the TNBS treatment was significant only on the 26th and 28th days (Figure 2). After the induction of the intestinal inflammatory process by TNBS, the tissue damage (estimated by scores) increased, already maximizing on the 2nd day, remaining high until the 10th day when it started dropping, becoming almost recovered on the 14th day. The relapse induced damage levels similar to those of the first treatment, peaking around day 20 and recovering on day 30. Adhesions, diarrhea, and the weight/length ratio presented similar behavior until the 14th day. However, after the relapse only the diarrhea percentage reached the high levels of the first treatment; the percentage of adhesions was about one fourth that of the first treatment, while the weight/length ratio showed very little alteration. Body weight gain was reduced by TNBS colitis from day 2 to day 6, the moment from which the animals started to gain weight again until the end of the experiment. However after the relapse of the inflammatory process, the animals showed loss of body weight again (Table 2). Statistical analyses show significant differences between noncolitic (saline) and colitic (TNBS) groups in relation to tissue damage, body weight, and colon weight/length ratio at days 8, 20, 26, and 28. For adhesions and diarrhea, the differences were significant at days 8, 20, and 26 but not on day 28 (Table 3).

**Figure 3:**
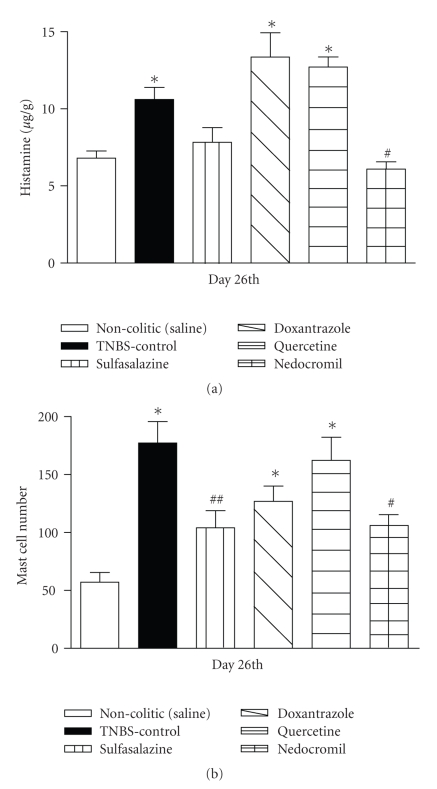
(a) Histamine concentration and (b) mast cell number in colon of Wistar rats with colitis induced by TNBS treated with sulfasalazine (25 mg/Kg/day) doxatranzole (5 mg/Kg/day), quercetine (10 mg/Kg/day), or nedocromil (100 mg/Kg/day) daily from 14th until 26th days of induction of the colitis. The results are expressed as mean ± SEM (*n* = 5–7). *P < .05 versus noncolitic group. ^*#*^ *P* < .05; ^*##*^ *P* < .01 versus TNBS control group (by ANOVA).

The colon histamine concentrations in colitic animals treated with doxantrazol or quercetin rose to about the same level as that of the TNBS control group, but were higher than those of the sulfasalazine, nedocromil, and noncolitic (saline) groups. There were no differences among the groups treated with sulfasalazine, nedocromil, and noncolitic (saline) (Figure 3(a)). The mast cell numbers in the colons of all these groups follow the same profile as the histamine concentration when compared with the noncolitic (saline) group (Figure 3(b)).

Pharmacological treatment of colitic rats with nedocromil decreased the colon histamine concentration compared with the TNBS control group (Figure 3(a)). Moreover, the treatments with nedocromil and sulfasalazine decreased the colon mast cell number compared with TNBS control (Figure 3(b)). 

The treatments with sulfasalazine, nedocromil, doxantrazole, and quercetin did not macroscopically attenuate the reactivation of the inflammatory process in the colonic tissue. There was no decrease in the macroscopic damage score or in the colonic weight/length ratio when compared with control colitic animals with relapse. In addition, no significant differences were found in the diarrhea incidence, in focal adhesions to adjacent organs, or in body weight (Table 4).

## 4. Discussion

Colitis induced in rats by the hapten TNBS has been widely used in investigating the physiopathology of this disease. However, this model has some limitations given that once TNBS has been administered intracolonically the inflammatory status resolves spontaneously with time until the colonic mucosa is completely healed, which is not the situation in human IBD [26]. Recently, Gálvez et al. [26] and Di Stasi et al. [27] reported a model of reactivated colitis similar to the protocol used in the present study. The second intracolonic admininstration of TNBS effectively resulted in a reactivation of the colonic inflammatory response, as evidenced by the alteration in the different macroscopic and general clinical parameters of inflammation. Intracolonic administration of TNBS/ethanol as originally described [21] elicited an inflammatory response in rats with characteristics similar to those reported elsewhere: experimental animals presented diarrhea [22] as well as anorexia and loss of weight [23], while their colonic segments appeared grossly ulcerated and inflamed, showing intense hyperemia and bowel wall thickening [28].

Histamine has frequently been used as a biochemical marker of mast cell numbers in tissues, because, other than in the rodent stomach, mast cells represent the major peripheral tissue repository of this amine [29]. Mast cell numbers are strongly correlated with tissue histamine levels in a number of diverse animal and human tissues, both in normal tissues and in those undergoing fibrosis or inflammation [29, 30].

In our current study, the colonic histamine concentration in colitic rats remained unchanged until 10 days after colitis induction and then started rising to a peak on day 26. Although the histamine concentration did not change until 10 days after colitis induction, the mast cell number increased both in the mucosal and submucosal layers. Already on day 8 it was about 10 times higher, and after that presented minor elevations until day 26. This increase was certainly due to mast cell proliferation; the absence of corresponding increase in the histamine concentration is on account of the immaturity of these mast cells, that consequently presented much less histamine in the cytoplasmic granules [31].

This increase of mast cells until day 26 corroborated previous findings obtained in the same model by Morris et al. [21] who mentioned that mast cell numbers were increased in inflamed tissues three and four weeks after colitis induction; however, the role of these cells was not specifically addressed. More recently, an increased mast cell count in the 5–20 day period [32] and an elevated mast cell protease-2 (RMPC-2) level at 3 weeks were reported in colonic tissue taken from TNBS rats [15].

In spite of the intense mast cell proliferation observed in the inflammatory process, these cells did not migrate to the spleen during recovery from the disease as observed in intestinal infection by *Trichinella spiralis* [17]. This fact suggests that mast cell proliferation can show different characteristics depending on the type of inflammatory process.

After inducing colitis by TNBS in Sprague Dawley rats, Gelbamann and Barrett [5] also did not observe differences in colonic histamine concentration until the end of the first week, but after 4 weeks this parameter had risen sharply. It is interesting to note that these studies did not employ relapse; however, after 28 days the histamine concentration in their experiments (16 *μ*g/g) was higher than in ours (10.5 *μ*g/g), suggesting that the relapse did not cause additional proliferation of mast cells. In contrast, the relapse caused extra stimuli that provoked tissue damage and higher percentages of adhesions and diarrhea, even though not much change could be seen in body weight and mass/length ratio.

Xu et al. [32] reported that the mast cell number in the colon of Sabra rats with TNBS-induced colitis was lower during the first five days after induction, suggesting mast cell degranulation. This effect was not observed in our experiments since mast cells were more numerous on day 8 compared with noncolitics rats (saline group), suggesting the absence of mast cell degranulation.

Quercetin, doxantrazole, and nedocromil cause mast cell stabilization with consequent inhibition of histamine release [33]. In the present study we have shown that pretreatment with either quercetin or doxantrazole did not change the histamine concentration in the colon, suggesting absence of histamine release during the intestinal inflammatory process; otherwise, these drugs would cause an increase in the histamine concentration. These treatments with quercetin or doxantrazole suggest that mast cells are not activated in the intestinal inflammatory process induced by TNBS. Similarly, Gelbmann and Barrett [5] also did not observe histamine concentration differences in the colon of colitic rats treated with another antihistaminic agent, diphenhydramine, when compared with TNBS-control rats.

Besides not increasing the histamine concentration, nedocromil causes a decrease in histamine and mast cell number, probably due to its inhibition of the proliferation and differentiation of these cells [32]. Similarly, sulfasalazine also causes reduction of mast cell number possibly due to its well-known anti-inflammatory effect [34].

In summary, we have shown that despite mast cells being associated with the intestinal inflammatory process as demonstrated by the elevations observed in mast cell number and histamine concentration, it seems that these cells and mediators do not interfere with colitis, at least when this inflammatory process was induced by administration of TNBS. These results are in agreement with Chin and Barrett [35] and Fukumoto et al. [15] who have demonstrated that mast cells are not essential to the development of TNBS-induced colitis in rats or mice. In addition, the proliferated mast cells do not migrate to the spleen during colitis remission, in contrast to what occurs in infection by parasites.

## Figures and Tables

**Table 1:** Criteria for assessment of macroscopic colonic damage.

Score	Criteria
0	No damage
1	Hyperemia, no ulcers
2	Linear ulcer with no significant inflammation
3	Linear ulcer with inflammation at one site
4	Two or more sites of ulceration/inflammation
5	Two or more major sites of ulceration and inflammation or one site of ulceration/inflammation extending >1 cm along the length of the colon
6–10	If damage covers >2 cm along the colon, the score is increased by 1 for each additional centimeter of involvement

**Table 2:** Damage score, changes in colonic weight, changes in body weight and incidence of adhesions and diarrhoea of colitic rats with relapse induced by TNBS.

Days of treatment	Damage score^(a)^ (0–10)	Colonic weight^(b)^ (mg/cm)	Body weight change (%)	Adhesions (%)	Diarrhoea (%)
0	0 (0–0)	67.2 ± 4.05	4.90 ± 0.50	0	0
2	7 (6–7.5)	129.0 ± 9.72	−7.20 ± 1.29	60	40
4	8 (6.5–9.5)	157.0 ± 17.35	−10.50 ± 0.52	80	100
6	7 (6–8)	196.8 ± 40.45	−3.56 ± 1.52	100	100
8	6 (5–7)	130.2 ± 9.38	15.98 ± 2.67	60	80
10	7 (3.5–8)	302.3 ± 63.78	24.02 ± 1.26	40	80
12	5 (1–5)	159.8 ± 16.09	23.78 ± 1.58	20	40
14	1 (1-1)	129.4 ± 6.01	26.48 ± 0.82	0	80
16	6 (6–6.5)	159.9 ± 18.21	10.52 ± 0.85	20	100
18	7 (6–8)	172.5 ± 17.37	14.68 ± 1.68	20	80
20	6 (5–6)	147.2 ± 9.59	24.95 ± 1.51	20	100
22	5.5 (5–6.5)	195.4 ± 17.77	29.12 ± 1.28	25	75
24	5 (5–6)	149.5 ± 15.01	30.02 ± 1.96	0	75
26	3 (2-2)	130.8 ± 4.97	35.85 ± 1.91	0	25
28	2 (2–3)	142.4 ± 3.76	50.02 ± 2.02	0	0
32	1 (1–4)	116.7 ± 6.89	51.58 ± 2.94	0	0
36	1 (0–1)	123.6 ± 5.89	56.44 ± 2.19	0	0
40	1 (0–1)	128.8 ± 3.23	62.85 ± 2.55	0	0

^(a)^Score data are expressed as median and interquartil interval. ^(b)^Body weight changes is expressed as percentage change from the start of the experiment (*N* = 6).

**Table 3:** Comparison between noncolitic and TNBS-control groups on damage score, colonic weight, body weight changes and incidence of adhesions and diarrhoea.

Group (*n* = 5–11) and days of treatment	Damage score^(a)^ (0–10)	Colonic weight^(b)^ (mg/cm)	Body weight change (%)	Adhesion (%)	Diarrhoea (%)
Noncolitic (saline) (day 8th)	0	85.5 ± 2.77	42.00 ± 3.93	0	0
TNBS-control (day 8th)	6 (5–7)*	130.2 ± 9.38*	29.15 ± 2.23*	60*	80*
Noncolitic (saline) (day 20th)	0	88.5 ± 2.42	41.70 ± 2.24	0	0
TNBS-control (day 20th)	6(5–6)*	147.2 ± 9.59*	34.18 ± 1.94*	20*	100*
Noncolitic (saline)(day 26th)	0	101.2 ± 6.22	53.56 ± 2.37	0	0
TNBS-control (day 26th)	3(2-2)*	130.8 ± 4.97*	46.20 ± 1.45*	50*	50*
Noncolitic (saline) (day 28th)	0	81.4 ± 1.98	57.55 ± 2.12	0	0
TNBS-control (day 28th)	2(2–3)*	142.4 ± 3.76*	51.61 ± 1.19*	0	0

^(a)^Score data are expressed as median and interquartil interval. ^(b)^Colonic weight data are expressed as mean ± S.E.M. *Groups differ significantly from the noncolitic group −*P* < .05.

**Table 4:** Effects of sulfasalazine, doxantrazole, quercetine, and nedocromil treatment on damage score, changes in colonic weight, body weight, and incidence of diarrhea and adhesions in reactivated TNBS colitis.

Group (*n* = 5–7) day 26th	Damage score^(a)^ (0–10)	Colonic weight^(b)^ (mg/cm)	Body weight change (%)	Adhesion (%)	Diarrhoea (%)
Noncolitic	0*	107.65 ± 6.80**	50.05 ± 2.45*	0*	0*
TNBS-control	5(3–6)	145.56 ± 3.03	40.58 ± 1.15	58	28.5
Sulfasalazine	1(1–4)	142.52 ± 5.77	45.02 ± 2.93	20	14.0
Doxantrazole	3(1–3)	132.50 ± 4.22	40.45 ± 2.58	58	28.5
Quercetin	2(1–3)	138.08 ± 3.00	44.49 ± 2.09	40	14.0
Nedocromil	1(1–3)	134.23 ± 5.82	46.78 ± 1.98	40	14.0

^(a)^Score data are expressed as median and interquartil interval. ^(b)^Colonic weight data are expressed as mean ± S.E.M. *Groups differ significantly from the TNBS group −*P* < .05. All groups differ significantly from the noncolitic (saline) group (*P* < .01, not shown).
